# Supplementary material for: Neural Interruption by Unilateral Labyrinthectomy Biases the Directional Preference of Otolith-Related Vestibular Neurons
Source: Brain Sci. 2021 Jul 26;11(8):987. doi: 10.3390/brainsci11080987 (PMC8393366; doi:10.3390/brainsci11080987)
Supplement: Supplementary file 1 [file brainsci-11-00987-s001.zip › brainsci-1261589-supplementary.pdf]

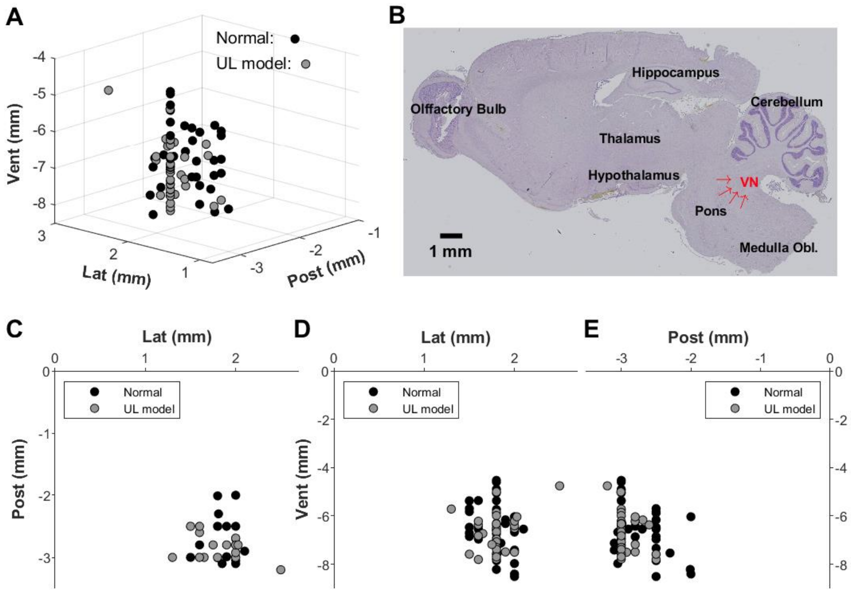

Figure S1. Neuronal recording area in the brain. (**A**) 3D area for neural recordings. (B) Sagittal section of the whole brain to identify Vestibular nucleus (VN) in red with arrows. (C) 2D area for neural recordings: lateral vs posterior direction from the center (D) 2D area for neural recordings: lateral vs ventral (E) 2D area for neural recordings: posterior vs ventral.

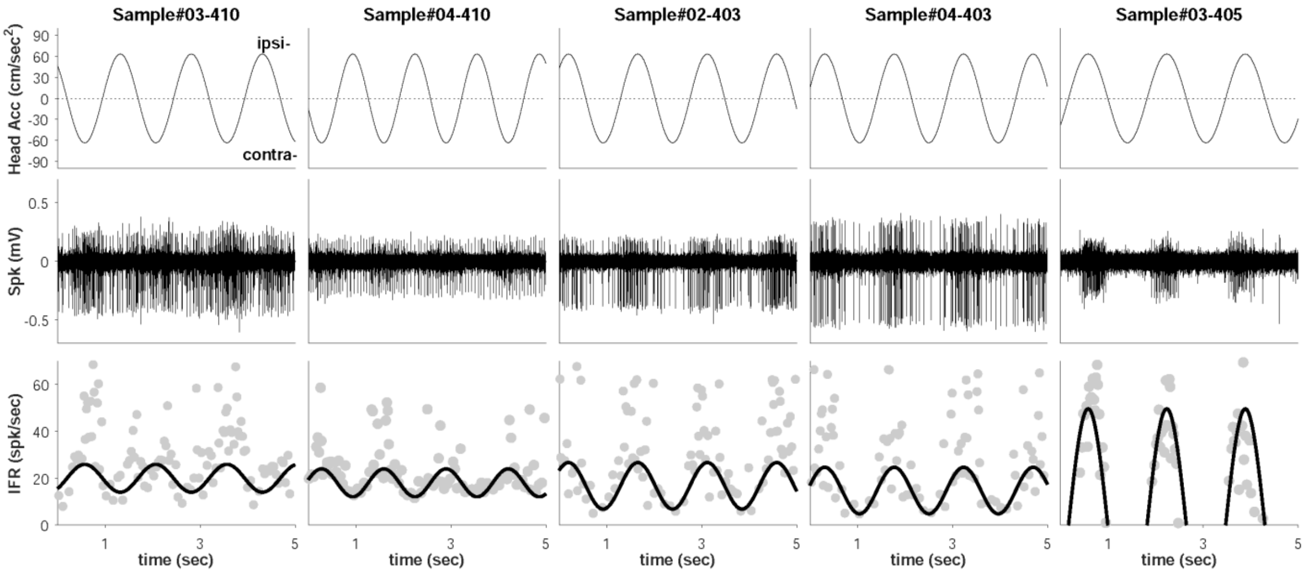

Figure S2. Extra examples of otolith-related neuronal responses during head acceleration. Two samples (#03-410 and #04-410) were identified as a contra-preferred response while others were ipsi-preferred. The first row showed head acceleration to stimulate the neuronal responses, and the second presented the neuronal spikes in mili-volts. The last one showed the relevant instantaneous firing rate and its curve-fitting.
